# Brief Report: Opioid‐related disorders increase patient‐directed discharge in admissions for hemorrhagic or ischemic stroke

**DOI:** 10.1111/ajad.70157

**Published:** 2026-03-29

**Authors:** Rohan M. Shah, Shiv Patel, Brent Schnipke, Brandon Hamm

**Affiliations:** ^1^ Northwestern University Feinberg School of Medicine Chicago Illinois USA

## Abstract

**Background and Objectives:**

Opioid use has been linked to increased stroke incidence and severity; however, data on effects during acute stroke hospitalization are limited.

**Methods:**

We used the 2016–2020 National Inpatient Sample to evaluate the effects of opioid disorders on patient‐directed discharge (PDD) and length of stay (LOS) in 3,101,545 patients.

**Results:**

On multivariate analysis, opioid disorders were independently associated with discharge AMA (aOR: 2.29; 95% CI: 1.98–2.65) and longer LOS (β: 1.5, SE: 0.138; *p* < .001).

**Discussion and Conclusions:**

Opioid disorders may predispose PDD and prolonged hospital courses in stroke admissions.

**Scientific Significance:**

Opioid disorders may warrant targeted inpatient management and health system planning in stroke.

## INTRODUCTION

Stroke remains one of the most significant causes of long‐term disability and mortality in the United States, affecting an estimated 800,000 individuals each year.^1^ At the same time, the opioid epidemic has yielded a dramatic increase in the prevalence of opioid use disorder (OUD), with one study estimating rates nearly doubled from 2008 to 2017.^2^ Per the Centers for Disease Control and Prevention (CDC), 6.1 million people aged 12 or older reported having an OUD in 2022, underscoring the scale of this national problem.^3^

Opioid use, especially in the setting of intravenous drug use, is a known possible contributor to stroke incidence and severity, secondary to increased likelihood of microvascular dysfunction, worse cardiovascular profiles, and infective endocarditis.^4^ However, there is still limited data quantifying effects of pre‐existing OUD on discharge disposition and acute hospitalization course immediately after stroke, a timeframe which is critical for recovery. In the present study, we examine whether patients with a history of opioid‐related disorders are more likely to discharge before medically advised and experience prolonged hospital length of stay (LOS) after admission for stroke, compared to counterparts.

## METHODS

We included all patients aged ≥18 with a primary diagnosis of ischemic or hemorrhagic stroke in the National Inpatient Sample (NIS) years 2016–2020. Patients were stratified into two groups based on the presence of a diagnosed opioid‐related disorder, determined using Clinical Classifications Software Refined code MBD018, provided by the NIS. The codes included within this schema encompass opioid use disorder, along with related diagnoses describing opioid abuse, dependence, or poisoning. The primary outcome was discharge prior to medically advised, commonly referred to as “patient‐directed discharge” (PDD). While the NIS variable uses the term “against medical advice,” we use PDD here to reflect the contemporary and increasingly preferred terminology. Hospital LOS was a secondary outcome. We conducted multivariate logistic and linear regressions to identify any independent effects of opioid use on outcomes. Models controlled for age, sex, race/ethnicity, household income by zip code, insurance status, and several comorbidities—hypertension, diabetes, obesity, malignancy, and lung disease. An *a priori* level of significance was determined at *p* < .05. Statistical analyses were conducted using R 4.4.0 (Vienna, Austria). This study used publicly‐available, de‐identified data and was therefore exempt from Institutional Board Review.

## RESULTS

Within 3,101,545 patients hospitalized for ischemic or hemorrhagic stroke, 27,770 (0.90%) had a concurrent diagnosis of opioid use disorder. Table 1 outlines the complete demographics of both patients with opioid‐related disorders and controls.

**TABLE 1:** Sample characteristics.

	Control cohort	Stroke patients w/OUD	
Characteristic	*N* = 3,073,775^a^	*N* = 27,770^a^	*p*‐Value^b^
**Age, mean (SD)**	69.6 (14.2)	59.4 (14.3)	**<.001**
**Age Group**			**<.001**
18–39 years	85,995 (2.8%)	2630 (9.5%)	
40–59 years	649,610 (21.1%)	11,195 (40.3%)	
60–79 years	1,455,480 (47.4%)	11,645 (41.9%)	
80+ years	882,690 (28.7%)	2300 (8.3%)	
**Sex**			.069
Male	1,552,045 (50.5%)	14,365 (51.7%)	
Female	1,521,730 (49.5%)	13,405 (48.3%)	
**Race**			**<.001**
White	2,064,115 (67.2%)	18,785 (67.6%)	
Black	529,980 (17.2%)	5655 (20.4%)	
Hispanic	270,960 (8.8%)	2160 (7.8%)	
Asian or Pacific Islander	107,385 (3.5%)	315 (1.1%)	
Native American	14,035 (0.5%)	185 (0.7%)	
Other	87,300 (2.8%)	670 (2.4%)	
**Median Income Quartile of ZIP Code**			**<.001**
76th to 100th percentile	599,995 (19.5%)	4085 (14.7%)	
0 to 25th percentile	942,585 (30.7%)	10,325 (37.2%)	
26th to 50th percentile	803,600 (26.1%)	7380 (26.6%)	
51st to 75th percentile	727,595 (23.7%)	5980 (21.5%)	
**Health Insurance**			**<.001**
Private insurance	603,330 (19.6%)	4315 (15.5%)	
Medicare	1,960,340 (63.8%)	13,140 (47.3%)	
Medicaid	296,515 (9.6%)	8170 (29.4%)	
Other	213,590 (6.9%)	2145 (7.7%)	
**Medical Comorbidities**			
Hypertension	2,624,530 (85.4%)	21,420 (77.1%)	**<.001**
Diabetes	1,137,065 (37.0%)	8620 (31.0%)	**<.001**
Obesity	428,775 (13.9%)	4230 (15.2%)	.006
History of cancer	157,380 (5.1%)	2005 (7.2%)	**<.001**
Lung disease	469,685 (15.3%)	7030 (25.3%)	**<.001**
**Mortality**			.072
Alive	2,865,420 (93.2%)	26,060 (93.8%)	
Died	208,355 (6.8%)	1710 (6.2%)	
**Length of Stay (μ, SD)**	5.7 (8.0)	7.9 (10.3)	**<.001**
**Cost of Care (μ, SD)**	$18,289.6 (25,408.8)	$25,623.7 (34,809.0)	**<.001**
**Patient Directed Discharge**	28,725 (1.0%)	950 (3.6%)	**<.001**

*Note*: The bold values indicate the statistical significance of *p* < 0.05.

^a^ *n* (%).

^b^ Design‐based Kruskal–Wallis test; Pearson's X^2: Rao & Scott adjustment.

Compared with controls, patients with opioid‐related disorders had more frequent PDD (3.6% vs. 1.0%; *p* < .001). On multivariate analysis, opioid‐related disorders were independently associated with PDD (aOR: 2.29; 95% CI: 1.98–2.65). Patients with opioid‐related disorders also had longer average hospital lengths of stay (7.9; SD 10.3) versus 5.7 (SD 8.0 days, *p* < .001). On linear regression, opioid‐related disorders were independently associated with longer LOS than patients without OUD (β: 1.5, SE: 0.138; *p* < .001).

## DISCUSSION

Our study provides evidence that the presence of comorbid opioid‐related disorders may increase the likelihood of PDD and lengthen hospital stays after stroke. Our study echoes previous research that suggests patients using opioids are at risk for PDD and provides data more specific to stroke patients.^5^, ^6^ Additionally, our study reports PDD rates in the contemporary fentanyl‐dominant synthetic opioid era, a period characterized by markedly more volatile use patterns and higher‐acuity presentations. For context, recent national data demonstrated that between 2016 and 2020, the proportion of PDD in opioid‐related disorders increased from 9.3% to 17.0%, underscoring that the newer opioid landscape may be even more prone to early patient‐initiated discharge.^7^

Opioid use may impact immediate post‐stroke recovery and disposition due to a multitude of factors such as withdrawal‐related challenges, decreased engagement in rehabilitation services (speech, physical, occupational therapies), possible financial/housing instability, limited caregiver support, and higher number of medical comorbidities.^8^ In addition, system‐level factors may further compound these barriers, including institutional stigma, limited availability of medications for OUD (MOUD), or exclusionary policies that restrict post‐acute care options for individuals with OUD.^9^

Systemic and interdisciplinary interventions, such as routine screening and integration of addiction consultation services, into emergency departments, urgent care, and inpatient settings can improve observed medical disparities in opioid‐using populations.^10^ MOUD may also decrease PDD rates, with an instructive study by Wang et al. demonstrating a decrease from 59.6% to 30.0% in patients receiving medications when admitted with opioid‐induced complications.^5^ Future research should evaluate if the use of MOUD reduces the likelihood of early discharge in stroke patients with OUD. Given the above‐mentioned rising rates of substance use in the United States and disproportionate effects on clinical trajectories, it is also critical for hospital systems to prioritize clinicians' readiness and training in identifying and managing substance use disorders in the inpatient setting.

Ultimately, our analysis highlights that opioid‐related disorders may be an independent risk factor for PDD in acute stroke hospitalizations, possibly predisposing disruptions on essential referrals to post‐acute rehabilitation, worsening functional recovery, morbidity, and mortality.

## CONFLICT OF INTEREST STATEMENT

The authors declare no conflicts of interest. The authors alone are responsible for the content and writing of this paper.
